# Understanding the Intransigence of Malaria in Malawi

**DOI:** 10.4269/ajtmh.21-1263

**Published:** 2022-10-13

**Authors:** Lauren M. Cohee, Jessy Goupeyou-Youmsi, Karl B. Seydel, Charles Mangani, Peter Ntenda, Alick Sixpence, Rex B. Mbewe, Alfred Matengeni, Shannon Takala-Harrison, Edward D. Walker, Mark L. Wilson, Themba Mzilahowa, Miriam K. Laufer, Clarissa Valim, Terrie E. Taylor, Don P. Mathanga

**Affiliations:** ^1^ Center for Vaccine Development and Global Health, University of Maryland School of Medicine, Baltimore, Maryland;; ^2^ Malaria Alert Centre, Kamuzu University of Health Sciences, Blantyre, Malawi;; ^3^ Department of Osteopathic Medical Specialties, College of Osteopathic Medicine, Michigan State University, East Lansing, Michigan;; ^4^ School of Public Health, Kamuzu University of Health Sciences, Blantyre, Malawi;; ^5^ Department of Physics and Biochemical Sciences, Malawi University of Business and Applied Sciences, Blantyre, Malawi;; ^6^ Department of Microbiology and Molecular Genetics, Michigan State University, East Lansing, Michigan;; ^7^ Department of Epidemiology, School of Public Health, University of Michigan, Ann Arbor, Michigan;; ^8^ Department of Global Health, Boston University School of Public Health, Boston, Massachusetts

## Abstract

Despite the scale-up of interventions against malaria over the past decade, this disease remains a leading threat to health in Malawi. To evaluate the epidemiology of both *Plasmodium falciparum* infection and malaria disease, the Malawi International Center of Excellence for Malaria Research (ICEMR) has developed and implemented diverse and robust surveillance and research projects. Descriptive studies in ICEMR Phase 1 increased our understanding of the declining effectiveness of long-lasting insecticidal nets (LLINs), the role of school-age children in malaria parasite transmission, and the complexity of host–parasite interactions leading to disease. These findings informed the design of ICEMR Phase 2 to test hypotheses about LLIN use and effectiveness, vector resistance to insecticides, demographic targets of malaria control, patterns and causes of asymptomatic to life-threatening disease, and the impacts of RTS,S vaccination plus piperonyl butoxide-treated LLINs on infection and disease in young children. These investigations are helping us to understand mosquito-to-human and human-to-mosquito transmission in the context of Malawi's intransigent malaria problem.

## INTRODUCTION

Malaria continues to be one of the greatest threats to health in Malawi, with the risk of *Plasmodium falciparum* infection existing throughout the country. Although interventions have been scaled-up over the past decade, malaria infection risk and morbidity remain high, with some reports showing increasing malaria burden.^1^ In 2020, the incidence of malaria illness ranged by district from 155 to 1,050 cases per 1,000 population.^2^ For a population of 17.6 million, 9.8 million courses of artemisinin-based combination therapy (ACT) were dispensed in 2020 alone.^2^^,^^3^ In 2011, the U.S. National Institutes of Health, through Michigan State University, established the International Center of Excellence for Malaria Research (ICEMR) at Kamuzu University of Health Sciences (formerly the University of Malawi College of Medicine) with the goal of creating a platform capable of designing, implementing, and evaluating malaria control and prevention strategies in Malawi. The projects aimed to understand infection burden and malaria disease determinants in both urban and rural areas (Table 1). In the context of these studies, we developed capabilities in grant and data management and molecular analysis. Results from these studies were descriptive in nature to establish a systematic analysis of malaria epidemiology in our setting and paved the way for more specific and hypothesis-driven evaluations in our second grant.

**Table 1:** Overview of Malawi ICEMR-supported studies

Study design (years)	Settings	Participants	Participant follow-up	Associated vector sampling
A. ICEMR Phase 1: Determinants of Malaria Disease in Malawi				
Characterize the changing epidemiology of malaria in diverse settings				
Facility-based surveillance (2012–2013)	Peri-urban Rural highland Rural lowland	All ages, presenting at health center	> 50,000 visits, 25,486 with a febrile illness	N/A
Household-based cross-sectional surveys (2012–2016)	Peri-urban Rural highland Rural lowland	All ages	16,650 visits over six surveys	Prokopack aspiration, CDC light traps
School-based cohort (2015)	Rural highland Rural lowland	5- to 15-year-old students	786 students; 2,888 visits	N/A
Community cohort (2014–2017)	Rural lowland	All ages after malaria diagnosis	120 participants; 1,062 visits	N/A
Evaluate patterns and drivers of malaria in “urban” settings				
Case–control (2012–2015)	Urban/peri-urban	Children under 5 years	850	Prokopack aspiration, CDC light traps
Transect (2015–2016)	Urban-to-rural continuum	Children under 5 years	1,400	Prokopack aspiration, CDC light traps
B. ICEMR Phase 2: The Intransigence of Malaria in Malawi: Understanding Hidden Reservoirs, Successful Vectors and Prevention Failures				
Epidemiology of malaria risk in Malawi: vector resistance, ITNS, and human behavior				
Community cohort (2019–2021*)	Rural—pyrethroid only LLINs Rural—PBO LLINs	6 months–15 years	1,883 participants; 16,900 active and 4,398 passive case detection visits	Prokopack aspiration, CDC light traps, human landing catches, larval sampling for resistance testing
Reservoirs of transmission: targets for malaria control interventions				
Community cohort (2019–2021*)	Rural—pyrethroid only LLINs Rural—PBO LLINs	All ages	804 participants; 6,759 active and 1,290 passive case detection visits	Prokopack aspiration, pyrethrum spray catches, CDC light traps
Community cohort (2021–present)	Rural—PBO LLINs	All ages	Ongoing	Pyrethrum spray catches, CDC light traps (planned)
Malaria pathogenesis: the spectrum from asymptomatic infection to life-threatening disease				
Community cohort (2021–present)	Rural—pyrethroid only LLINs Rural—PBO LLINs	2–8 years	Five cohorts, 200 participants each Ongoing	N/A
Case–control (2021–present)	Referral hospital and rural-to-urban catchment areas	Cases: severe malaria Controls: age- and gender-matched in case village	25 cases, 23 controls Ongoing	N/A
VANSS: combined effects of RTS, S vaccination and PBO nets on malaria infection and transmission in Malawi				
Community cohort (2020)	Rural—pyrethroid only LLINs Rural—RTS,S vaccine + PBO LLINs	7–18 months (index children, age eligible for RTS,S vaccine 19 months–10 years (siblings)	919 (index + siblings) in pyrethroid-only LLINs 773 (index + siblings) in RTS,S/PBO	CDC light traps

ICEMR = International Center of Excellence for Malaria Research; LLIN = long-lasting insecticidal nets; N/A = not applicable; PBO = piperonyl butoxide; VANSS = Vaccine and Nets Study.

* Study activities halted due to COVID April 2020–April 2021.

In 2017, ICEMR Phase 2 funding was awarded for 7 years, during which our focus shifted toward understanding why and how malaria has remained intransigent in Malawi. These ongoing projects combine measurement of *Anopheles* vector abundance and behavior, *Plasmodium* infections of vectors and humans, and behavioral risk factors for transmission and disease. Using several longitudinal cohorts, we aim to understand population-level factors that affect malaria prevention and control interventions as well as individual-level host and parasite contributions that influence the spectrum of outcomes, ranging from asymptomatic infection to uncomplicated malaria illness and severe malaria disease (Table 1). In this report, we present scientific findings from projects that have taken place since the start of the Malawi ICEMR Program and discuss the implications of the research findings for malaria control in Malawi and the region. Specific policy impacts are described in the companion article by Mangani et al.^4^

## EPIDEMIOLOGY OF *P. FALCIPARUM* INFECTION AND MALARIA DISEASE IN MALAWI

Although there are decades of studies on clinical malaria and malaria-related mortality, little was known about asymptomatically infected people when the ICEMRs were launched in the early 2010s. ICEMR Phase 1 sought to describe the epidemiology of all *P. falciparum* infections and malaria disease using standardized approaches to enhanced facility-based surveillance, repeated cross-sectional surveys, and a cohort study (Table 1). We expanded surveillance to all age groups, in contrast to routine Malaria Indicator Surveys, which only focus on children under 5 years of age, and used molecular tools to detect submicroscopic infections. Across these studies, we described high prevalence of asymptomatic infections, evaluated the implications of asymptomatic infections on our understanding of malaria disease and transmission, as well as described the maintenance of sulfadoxine-pyrimethamine (SP) resistance despite changes in drug policy.

### The majority of *P. falciparum *infections are asymptomatic and prevalence peaks in older children.

**Figure 1.**
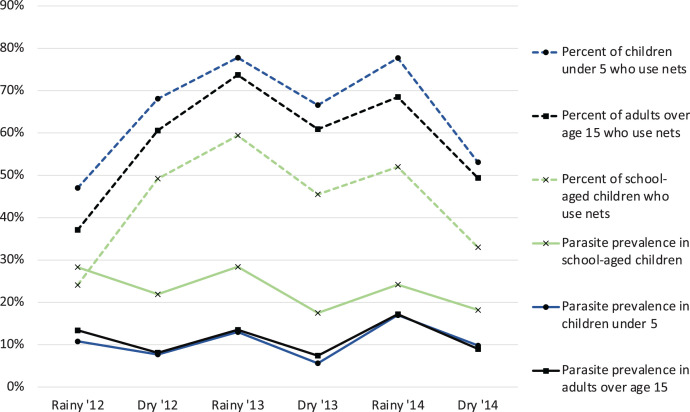
Infection prevalence and net use by age group. Vertical line indicates timing of national net distribution campaign. Previously published in Walldorf et al.^6^

From our surveys, 90% of infections were asymptomatic when defined as lack of fever in the past 48 hours.^5^ Independent of the transmission setting, parasite prevalence peaks in school-age children (age 6–15 years) (Figure 1).^5^^,^^6^ These findings are consistent with others across the sub-Saharan region and provide the foundation for subsequent and ongoing studies described here.

### The large pool of individuals with asymptomatic *Plasmodium* infections compromises the diagnosis of other etiologies of febrile illness.

In 2012, soon after Malawi began using malaria rapid diagnostic tests (mRDTs), we conducted 2 years of surveillance for malaria illness in health facilities in southern Malawi. Across all sites and in both the rainy and dry seasons, use of mRDTs was widespread with over 80% of patients with any symptoms of malaria having a mRDT test performed. One-quarter of tests were positive, implying a high burden of disease.^7^ However, a positive mRDT in the setting of fever in a high transmission setting where asymptomatic infections are common does not prove that the fever is due to malaria infection. Indeed, school-age children had the highest rate of positive mRDT results in our facility-based surveillance, but our cross-sectional studies indicate that many of these infections are asymptomatic, suggesting that other intercurrent illnesses are causing fever and the detection of *Plasmodium* infection may be incidental.

This finding highlights the need for a clinical algorithm that includes evaluation for other causes of fever even in the presence of a positive mRDT as well as diagnostics for other common causes of fever.

### Asymptomatic infections do not often progress to clinical disease.

The majority of infections detected in community-based surveillance are submicroscopic, and these low-density infections are not associated with fever.^8^ However, a small proportion of infections causes clinical disease. Through a longitudinal cohort of adults and children at a rural health center, we demonstrated that asymptomatic parasitemia was present in 23% of all visits. Of clinical episodes diagnosed by RDT that were preceded by asymptomatic infection and for which genotyping was performed, 92% of cases had a new *Plasmodium* genotype at the time of clinical presentation. Genotypes at time of clinical presentation were often mixed (both a persistent genotype and a new genotype) suggesting that although the presence of a persistent asymptomatic infection does not proceed to clinical disease, it also does not protect against the acquisition of an infection that leads to clinical disease.^9^ Ongoing studies described here will further evaluate host and parasite factors that determine the outcome of infections along the spectrum from asymptomatic infection to severe disease.

### SP resistance is maintained after changes in drug policy.

After a change in Malawi’s first-line therapy from chloroquine to SP in 1993, parasites regained chloroquine sensitivity within 8 years.^10^ The return of chloroquine sensitivity likely stemmed from a re-expansion of diverse chloroquine-susceptible parasites that survived drug pressure and expanded after the drug policy change, being more fit than drug-resistant parasites in the absence of chloroquine use.^11^ Malawi changed from SP as first-line therapy to artemether-lumefantrine (AL) in 2007, at which time the prevalence of highly SP-resistant “quintuple mutant” parasites was > 95%. In 2012, 5 years after the drug policy change, the prevalence of SP-resistant parasites remained high in Blantyre and rural sites with higher malaria transmission.^12^^,^^13^ Likewise, the characteristics of the corresponding selective sweeps in the genomic region harboring the drug-resistance genes showed little change over time.^12^ These results are consistent with SP-resistance mutations conferring little to no fitness cost to the parasite in the absence of drug pressure in this setting. Alternative explanations for the maintenance of SP resistance include fixation of SP-resistance mutations in the parasite population or continued drug pressure from intermittent preventive therapy in pregnant women or other populations. Thus, in contrast to chloroquine, resistance may jeopardize the future use of SP in Malawi. As concern for artemisinin and partner drug resistance increases in Malawi and throughout sub-Saharan Africa, surveillance networks such as ICEMR are critical frameworks to identify and monitor emerging resistance.

## INTERRUPTING *P. FALCIPARUM* TRANSMISSION FROM HUMAN HOST TO MOSQUITO: SCHOOL-AGE CHILDREN ARE KEY RESERVOIRS OF TRANSMISSION AND APPEALING TARGETS FOR NEW INTERVENTIONS

Throughout both phases of our ICEMR grants, we have evaluated the burden of malaria in school-age children and described their disproportionate contribution to perpetuating transmission.^14^^,^^15^ Results from our initial studies were limited by their cross-sectional design. In ICEMR Phase 2, we conduct more intensive investigations using longitudinal studies and sophisticated molecular approaches. Our results are informing the next steps in interventions to address infections in school-age children as a central approach to improving malaria control in Malawi.

### The peak prevalence of infection in school-age children has both biological and sociobehavioral underpinnings.

School-age children acquire new infections and maintain higher density infections like younger children. However, school-age children have acquired some immunity to clinical malarial disease and hence experience fewer symptoms and thus do not receive treatment to clear infections.^16^ This age group also benefits less from currently available prevention and treatment interventions: school-age children are least likely to sleep under bed nets, even when nets are available^5^^,^^6^^,^^17^^,^^18^ and are least likely to access prompt diagnosis and effective treatment.^19^ Low levels of net use among school-age children are often due, in part, to intrahousehold net allocation which prioritizes children under 5 years, pregnant women, and other adults, such as visitors. When school-age children reach adolescence, they often go to sleep later at night and may have sleeping spaces that are less amenable to hanging bed nets. Barriers to accessing malaria treatment among school-age children included inconvenient service hours at health facilities, long distances to health facilities and waiting time, restrictions and fear of COVID-19, stock-outs of drugs, poor provider attitudes, and fears around malaria testing and witchcraft (ICEMR, unpublished). Among the policy implications of these findings is that malaria control efforts should more carefully consider school-age children’s behavior regarding long-lasting insecticidal nets (LLINs) and that prompt treatment of malaria-like symptoms is needed to better meet their special needs. However, because most infections in this age group are subclinical, additional interventions, such as intermittent preventive treatment, may be needed to decrease the prevalence of infection in this age group.

### Infections in school-age children are also key reservoirs of human-to-mosquito transmission.

**Figure 2.**
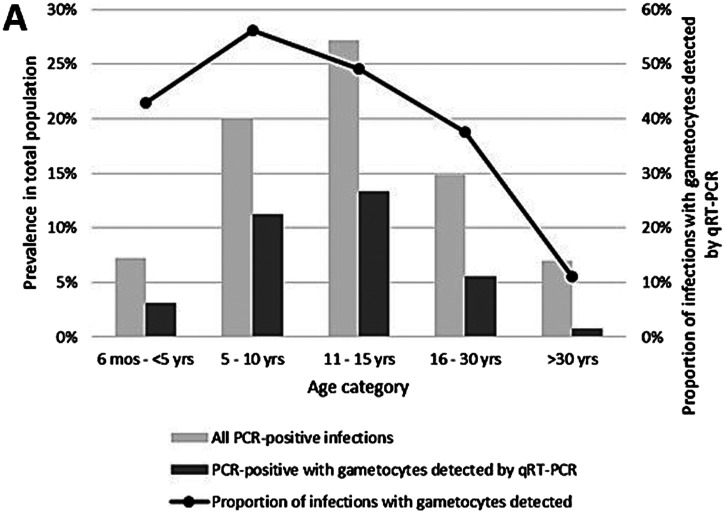
Distribution of gametocyte containing infections by age group: prevalence in the total population and proportion of infections. Previously published in Coalson et al.^14^

**Figure 3.**
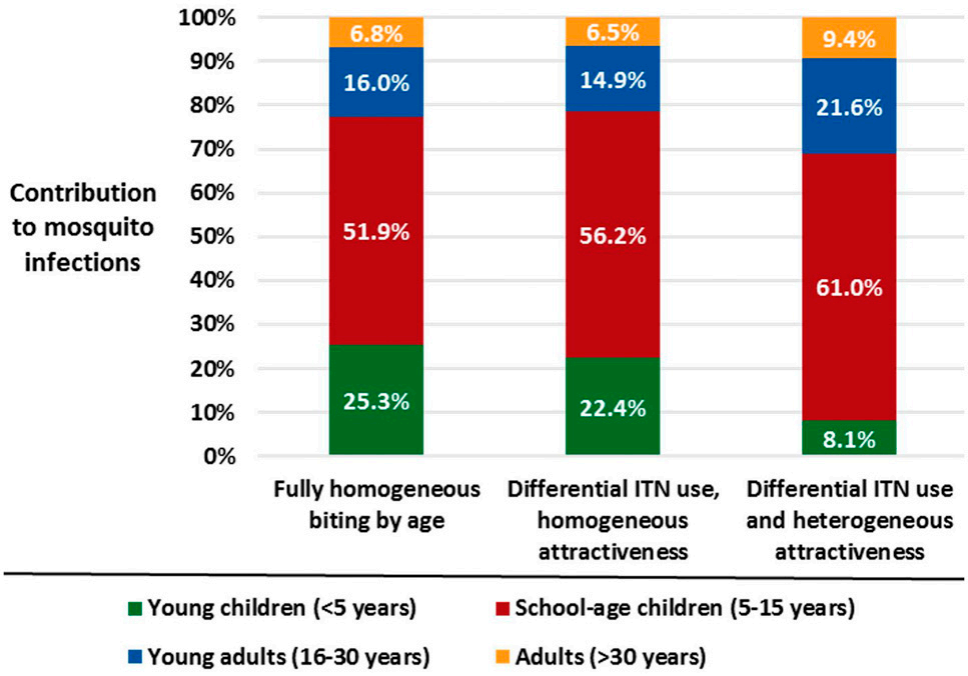
Age group contribution to mosquito infections in the rainy season using simulation models considering different estimates of vector-human interactions. Previously published in Coalson et al.^15^

Compared with younger children and adults, a higher proportion of infections in school-age children contain gametocytes, the parasite stage required for human-to-mosquito transmission (Figure 2).^14^ Because the prevalence of infection peaks in school-age children and a higher proportion of those infections contain gametocytes, nearly half of gametocyte-containing infections in the community are found in school-age children, who comprise only a third of the population in our study sites.^20^ Simulation models combining these data with estimates of availability of school-age children to biting *Anopheles* vectors based on LLIN use and body surface area suggest that school-age children are responsible for more than 60% of mosquito infections perpetuating transmission (Figure 3).^15^ These results imply that focusing transmission reduction interventions on school-age children will lead to population-level benefits.

### With increasing primary school enrollment across sub-Saharan Africa, schools are viable platforms to reduce the burden of malaria in school-age children.

In our study sites, 82% to 99% of 6- to 15-year-old children attend primary school.^20^ “School health days” currently deliver health interventions, such as deworming medications, to schoolchildren and are amenable to adding malaria control interventions.^21^ Our school-based cohort studies show that screening students for infection and treating those who are positive can halve the prevalence of infection and reduce anemia by nearly one-third.^22^ Furthermore, using the age distribution of gametocyte burden in the communities surrounding the schools, we estimate that implementing school-based screening and treatment could substantially reduce transmission in the community.^20^

## INTERRUPTING *P. FALCIPARUM* TRANSMISSION FROM THE MOSQUITO TO THE HUMAN HOST: UNDERSTANDING MOSQUITO BEHAVIOR AND INTERVENTION EFFECTIVENESS WILL HELP IDENTIFY MORE SUCCESSFUL APPROACHES FOR VECTOR CONTROL

LLINs and indoor residual spraying (IRS) are core vector control interventions recommended by WHO in all malaria-endemic settings. LLINs constitute the most widely used vector control intervention and remain the cornerstone of malaria control in Malawi. Since 2011, three LLIN mass distribution campaigns have been conducted. These are supplemented by continuous distribution through antenatal and immunization clinics. Despite this massive distribution of LLINs, the decline in the burden of malaria has plateaued, and the risk of disease remains high.^1^ Persistent malaria transmission despite LLIN distribution could be due to net failure and/or limited net use. Thus, a primary focus of our ICEMR has been to evaluate interrelated factors that limit the effectiveness of LLINs, including net availability; proper, consistent use; net deterioration; vector abundance and behavior; and the susceptibility of vectors to insecticides. Our ongoing studies focus on the impact of the distribution of LLINs containing pyrethroids and the synergist piperonyl butoxide (PBO nets) in Machinga District one of our ICEMR sites in 2018 and IRS in Balaka District, our other ICEMR site, with clothianidin in 2020 and clothianidin and deltamethrin in 2021.

### Failure to use LLINs occurs for a wide range of reasons even when nets are available.

In Malawi, mass LLIN distribution campaigns have historically been conducted every three years. Following each net distribution campaign, overall net ownership peaks near 90% and reported use approaches 80% (ICEMR unpublished).^17^ However, net ownership and net use decline between distribution campaigns. We aim to identify barriers to reaching and maintaining higher levels of ownership and use. Predictors of net ownership in our setting include household distance from health centers^23^ and household education and wealth. As described earlier, age is a key predictor of net use. In addition, our qualitative studies suggest that nonuse is driven by physical challenges (e.g., hanging rectangular nets compared with round nets), discomfort in using nets (e.g., irritation from insecticides, heat), bedbug infestation, and conspiratorial misconceptions about nets (e.g., nets facilitate bewitching) (Mangani et al., in preparation). These findings suggest that improved continuous distribution systems, improved net designs, and behavior change communication strategies could improve net use.

### Rapid deterioration of LLINs after distribution campaign.

The deterioration in net condition (both physical integrity and insecticide potency) over time can affect the LLIN effectiveness. After the 2012 distribution campaign, we assessed how physical integrity and age impact net effectiveness.^24^ Net age, as an indicator of insecticide potency, was associated with decreased net effectiveness; Sleeping under a LLIN that was more than 2 years old was associated with 50% increased odds of *P. falciparum* infection compared with those using a newer LLIN. Another study in Malawi demonstrated that among individuals 1 to 2 years old, there was an 18% reduction in malaria risk in users of LLINs with no holes (of any size) compared with users of LLINs with at least one hole.^25^ Thus, both insecticide potency and physical integrity contribute to declines in LLIN effectiveness after distribution. Improved LLIN design (fabric integrity and insecticide choice) and/or increase frequency of distribution campaigns can address these factors.

### Pyrethroid resistance requires new approaches to vector control.

Malaria vector control in Malawi relies primarily on use of LLINs, and because LLINs may be compromised in the face of pyrethroid resistance, there are concerns that the high burden of disease in Malawi could be due to LLIN failure from high insecticide resistance. Insecticide resistance, largely to pyrethroid insecticides, is intense and widespread in Malawi.^26^^–^^30^ In our ongoing ICEMR studies, we monitor the susceptibility of vectors to permethrin, deltamethrin, pirimiphos-methyl, chlorfenapyr, and clothianidin. Our data confirm that *Anopheles* vector populations are resistant to pyrethroids (permethrin and deltamethrin) and only susceptible to pirimiphos-methyl, chlorfenapyr, and clothianidin. These findings are consistent with reports from across multiple sites in the country^31^ and contribute to a robust and comprehensive dataset that has informed the newly developed insecticide resistance management plan for malaria control in Malawi. As both IRS and nets containing pyrethroids combined with novel active ingredients are implemented in our ICEMR sites, we are monitoring epidemiologic and entomologic impacts including both resistance and resistance mechanisms. This will allow us to investigate whether the modeled impacts of these interventions are observed in practice and provide information on the potential for cross-resistance.

### Interventions impact vector species distribution and behavior.

Our work has shown that the malaria vector landscape in Malawi is currently dominated by *Anopheles funestus *s.s. and *An. arabiensis* in terms of disease transmission, population abundance, and geographic distribution.^30^^,^^32^ This represents a significant shift from the situation reported a decade ago when *An. gambiae *s.s. was the most important malaria vector species in the country.^33^ To investigate the impact of insecticides on vector abundance and behavior, we conducted indoor and outdoor human landing catches from September 2020 to March 2021, including the period of the 2020 IRS campaign in Balaka district (Kumala et al., unpublished data). Preliminary analyses show that *An. gambiae *s.l. largely exhibits outdoor biting behavior in both districts before and after the IRS campaign. However, *An. funestus *s.l., a vector that was largely indoor biting before the start of 2020 IRS campaign, exhibited increased outdoor biting behavior after IRS; this behavior gradually reversed over time. Results also show that the absolute abundance of both Anopheles species dropped sharply right after the IRS and gradually increased over the rainy season. Changes were also observed in the relative abundance of both vectors in Balaka district where An. *funestus *s.l. were progressively replaced by *An. gambiae s.l.*, from the dry to the rainy season. The hourly biting rates showed that both species, in the two districts (Machinga and Balaka), had a peak of activities between 2 and 4 am, a time of night when most people are sleeping. These results suggest that *An. funestus *s.l., known as a highly anthropophilic and indoor biting vector species, reduced in abundance due to the insecticide and concomitantly developed a tendency to bite outside. When the effect of the insecticide decreased, this species switched back to its natural indoor biting behavior. As for *An. gambiae *s.l., this species was not heavily affected by IRS because it is able to bite both indoor and outdoor and feeds on humans and other animals. Ongoing studies and further analyses of available data will determine which *Anopheles* species of the *gambiae* complex and *funestus* group are involved and clarify whether the change in the relative abundance of *An. funestus *s.l. over time might also be due to the change in climatic conditions.

### In the context described here, PBO nets are more effective than conventional nets, but the effects still wane over time.

**Figure 4.**
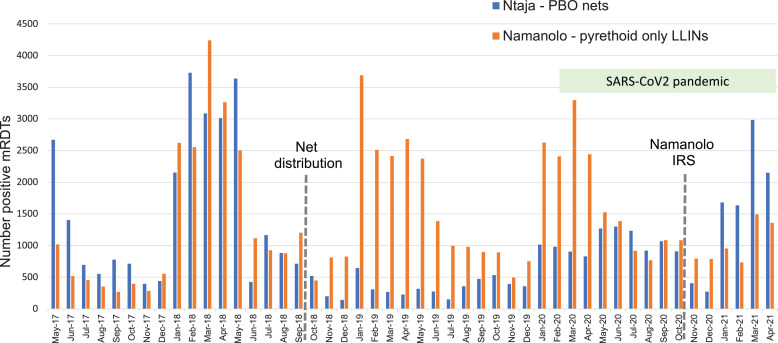
Monthly malaria cases in relation to intervention type and timing.

To address concerns about the impact of increasing insecticide resistance on LLIN effectiveness, the National Malaria Control Program distributed PBO nets to some districts during the 2018 campaign. One of our ICEMR sites (Machinga District) received PBO nets, whereas our other site (Balaka District) received pyrethroid-only LLINs. Our ongoing studies in ICEMR Phase 2 are evaluating the impact of this net distribution campaign on malaria disease and infection as well as entomologic indicators of transmission. Before the campaign, the health centers serving these sites had similar numbers of monthly malaria cases. After the mass distribution, there was a dramatic decrease in malaria cases in sites that received PBO nets (Figure 4). However, 1 year after the mass distribution campaign, the number of cases in the area that received PBO nets began to rise and approached precampaign levels after 3 years, indicating that the protection conferred by PBO nets wane over time similar to what has previously been demonstrated with pyrethroid-only LLINs. Analysis of the comparative impact on *P. falciparum* infection and transmission reservoirs is forthcoming.

Entomologic evaluations also demonstrate some impact of PBO nets, but transmission is sustained. Analysis of blood host selection patterns of the major vectors of human malaria in Malawi (*An. arabiensis*, *An. gambiae*, and *An. funestus*) reveal high rates of feeding on humans regardless of deployment of LLINs containing the synergist PBO or LLINs without this synergist.^32^ However, among all female *Anopheles*, a lower proportion blood-fed in Machinga district compared with Balaka, suggesting that PBO nets reduced successful blood-feeding (Goupeyou-Youmsi et al., in preparation). Infection rates in these vector populations for *P. falciparum*, as assessed using molecular detection, was a combined 15.6% resulting in an estimated entomological inoculation rate of ca. 0.09 infectious bites per person per night. Together with the health center data, these data suggest that PBO nets are more effective than pyrethroid-only LLINs, but, similar to pyrethroid-only LLINs, their effectiveness wanes over time. Our ongoing data analysis should reveal insights into why this waning effectiveness occurs.

## CHARACTERIZING THE HOST AND PARASITE DETERMINANTS OF THE OUTCOME OF *P. FALCIPARUM* INFECTION WILL DETERMINE HOW RESERVOIRS ARISE AND ARE SUSTAINED, IMPROVE OUR INTERPRETATION OF DISEASE DIAGNOSTICS, AND INFORM DEVELOPMENT OF VACCINES AND TREATMENTS

Although the majority of *P. falciparum* infections are asymptomatic, some infections result in a broad spectrum of disease from uncomplicated clinical malaria to severe disease and malaria-related mortality. The interactions that shift an infection that was previously in equilibrium (i.e., long term and asymptomatic) to clinical disease remain a major research interest. Is the introduction of *any* new parasite clone sufficient to disturb this relationship? Is the immune response somehow “primed” to move to clinical disease if another parasite clone is already present? Is there a certain subset of parasites that will always lead to clinical disease? Our previous work focused heavily on differentiating infections causing uncomplicated clinical malaria from those causing cerebral malaria as well as differentiating disease severity within cerebral malaria. In our ongoing ICEMR, we broadened our focus to include elucidating the host and parasite factors that lead to a broader spectrum: from asymptomatic infection to uncomplicated clinical malaria and severe disease. Elucidating the host and parasite factors that determine the clinical consequences of infection should help appropriately target malaria control efforts, as well as generate immunologic and pathogenesis insights relevant to the vaccine and adjunctive treatment options.

### Parasite contributions to disease severity.

PfEMP1 is the major family of parasite-produced proteins responsible for the binding of infected erythrocytes to vascular endothelial cells, a phenomenon that leads to microvascular obstruction and subsequent organ damage. In research completed at the ICEMR-funded laboratory, we have shown that the expression of the EPCR-binding subtype of the protein PfEMP1 is associated with the deadliest form of cerebral malaria, a disease that includes brain swelling.^34^ Expression of PfEMP1 variants will be further investigated in the ongoing ICEMR studies comparing children with asymptomatic infection and those with cerebral malaria.

Beyond PfEMP1, our group has used genomic approaches to identify possible targets of allele-specific malaria immunity. In high malaria transmission areas, individuals acquire immunity to clinical disease after repeated exposure to *P. falciparum* infections. This clinical immunity is thought to require a repertoire of immune responses to multiple alleles of diverse parasite antigens. However, not all these responses are protective. Using longitudinal samples from symptomatic participants to differentiate protective and nonprotective responses, we evaluated changes in parasite allele frequencies in individuals over time. This approach allowed identification of subdominant, but protective, epitopes that might otherwise be difficult to detect in immunological studies because they are masked by responses to immunodominant, but not protective, loci. Using whole genome sequence data generated from 140 *P. falciparum* isolates, we identified 25 genes that encode possible targets of allele-specific acquired immunity to clinical malaria.^35^ As part of future studies, we will further characterize these parasite proteins for their potential as vaccine candidates through immunological epitope mapping and functional studies of antibodies elicited to these proteins.

### Host contributions to disease severity.

In addition to heterogeneity in protein expression on the parasite, heterogeneity in the host response to parasites could explain the development of varying clinical states. Previous work has shown that a wide range of different genes are transcribed when the same host has an episode of uncomplicated or cerebral malaria.^36^ However, levels of T-cell subsets^37^ or seroreactivity to PfEMP1 genes^38^ do not differ between these two groups. Given the low level of conversion of asymptomatic parasitemia to clinical disease, theses syndromes appear to be distinct clinical states. Our current studies are designed to characterize both host and parasite factors that distinguish children with cerebral malaria from those with asymptomatic infection. Samples collected from these cohorts are currently being analyzed for differences in parasite protein expression, PfEMP1 expression, PfEMP1 seroreactivity, and characterization of chemokine, natural killer cell, and T-reg subsets. Differences not only will lead to insights into pathogenesis, but also could inform possible vaccine design.

## SUMMARY AND FUTURE PLANS

The Malawi ICEMR has been testing hypotheses about *Plasmodium* transmission and infection patterns and pathways that operate at various functional scales (regional, village, household, individual, cellular, and molecular) in an effort to understand and improve malaria control in Malawi. The first phase of our ICEMR generated key insights into the role of school-age children in *Plasmodium* transmission, the declining effectiveness of LLINs, and the complex host–parasite interactions leading to the severity of disease. These insights generated hypotheses for the recent and ongoing studies reported here, including evaluating the impact of PBO-treated LLINs and IRS. In addition, we are deepening understanding of *Plasmodium* transmission dynamics by assessing infectiousness of humans, the role of transmission-reducing immunity, and patterns of human–vector interactions. Results of these studies will inform development of interventions to decrease transmission and improve targeting of existing approaches. Examples of innovative strategies we are pursuing include school-based chemoprevention studies and implementation research to develop and evaluate an electronic vaccination registry to improve the cost-effectiveness of RTS,S vaccination of young children. Our ongoing cohort and case–control studies will define specific host and parasite factors that determine whether infections are asymptomatic, lead to clinical illness, or result in severe disease. These results should help develop prevention and therapeutic targets to decrease the most life-threatening cases of malaria infection. The diversity of basic and applied investigations at multiple levels of biological organization, involving questions and methods from epidemiology, ecology, parasitology, and immunology, will be refined and focused on future investigations as we continue to develop evidence to control disease and eliminate infection in Malawi.
